# The State of High‐Resolution Imaging of the Human Inner Ear: A Look Into the Black Box

**DOI:** 10.1002/advs.202500556

**Published:** 2025-06-05

**Authors:** Shelley Batts, Nancy Pham, Guillermo Tearney, Konstantina M. Stankovic

**Affiliations:** ^1^ Department of Otolaryngology – Head and Neck Surgery Stanford University School of Medicine 801 Welch Rd Stanford CA 94304 USA; ^2^ Department of Radiology Stanford University School of Medicine 1201 Welch Rd Stanford CA 94304 USA; ^3^ Department of Pathology Harvard Medical School 25 Shattuck Street Boston MA 02115 USA; ^4^ Wellman Center for Photomedicine Massachusetts General Hospital 55 Fruit St Boston MA 02114 USA; ^5^ Department of Neurosurgery Stanford University School of Medicine 300 Pasteur Dr Stanford CA 94304 USA; ^6^ Wu Tsai Neurosciences Institute Stanford University 288 Campus Drive Stanford CA 94305 USA

**Keywords:** clinical imaging, cochlea, endoscopy, hair cell, sensorineural hearing loss, spiral ganglion neuron

## Abstract

Unlike most medical fields, otology has not benefited from the transformative impact of high‐resolution, cellular‐level imaging. The sensorineural cells required for human hearing—located within the cochlea—are just 10–50 µm, placing them outside the resolution of magnetic resonance imaging, computed tomography, and positron emission tomography. These cells are highly mechano‐ and chemo‐sensitive, and their death or dysfunction underlie the vast majority of hearing loss. Further, the cochlea is only 4–7 mm in diameter, has complex anatomy, and is deeply embedded in bone. Cochlear blood flow is partially separated by a blood barrier, limiting access to radiotracers or fluorophores. These and other features have left the human cochlea as a “black box” that cannot be assessed with high precision in vivo, limiting the development of novel hearing loss therapies. The benefits and drawbacks of existing medical imaging techniques used to diagnose disorders of the human inner ear are discussed, as well as those of emerging technologies that may help overcome challenges to access, resolution, and functional detail. A comprehensive and up‐to‐date discussion is provided on research efforts to improve and adapt current clinical imaging methods and introduce recent innovations that have shown exciting promise for deriving both structural and metabolic information from cochlear cells.

## Graphical abstract

High‐resolution imaging of the cochlea is challenged by numerous unique aspects of this organ, where sensorineural cells, crucial for hearing, are too small for conventional modalities like magnetic resonance imaging and computed tomography. This article reviews current imaging techniques, emerging technologies, and innovations aimed at improving cellular‐level assessment of the inner ear, highlighting future directions for clinical application.

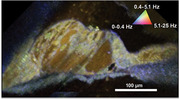

## 1 Introduction

Beginning from the invention of the compound light microscope in the 16^th^ century, advances in imaging technology have greatly benefited patients by improving the accuracy of medical diagnoses and enhancing the planning and performance of surgical procedures.^[^ ^1^ ^]^ In particular, the development of X‐ray in the 19^th^ century catalyzed a new era of non‐invasive medical imaging in the 20^th^ century, including ultrasound, tomography techniques, and magnetic resonance imaging (MRI).^[^ ^2^ ^]^ Equally pivotal was the introduction of monocular and binocular optical microscopy to surgical settings by otologic surgeons Carl Olof Nylén and Gunnar Holmgren, respectively, in the early 1920s.^[^ ^3^ ^]^ Since then, binocular microscopy has galvanized progress in all branches of surgery, including microvascular reconstruction, organ transplantation, neurosurgery, ophthalmology, and beyond. Both non‐invasive and direct imaging technologies have been further refined over time to provide ever‐increasing resolution, probe deeper into the body's structures, and, in some cases, offer functional as well as structural information. Contrast agents and radioisotopes have been employed to highlight specific tissues or dynamic processes among adjacent structures.^[^ ^4^ ^]^ In parallel, minimally invasive imaging modalities such as endoscopy and microendoscopy have become powerful tools for overcoming limitations related to imaging access or penetration depth.^[^ ^5^ ^]^

**Figure 1:**
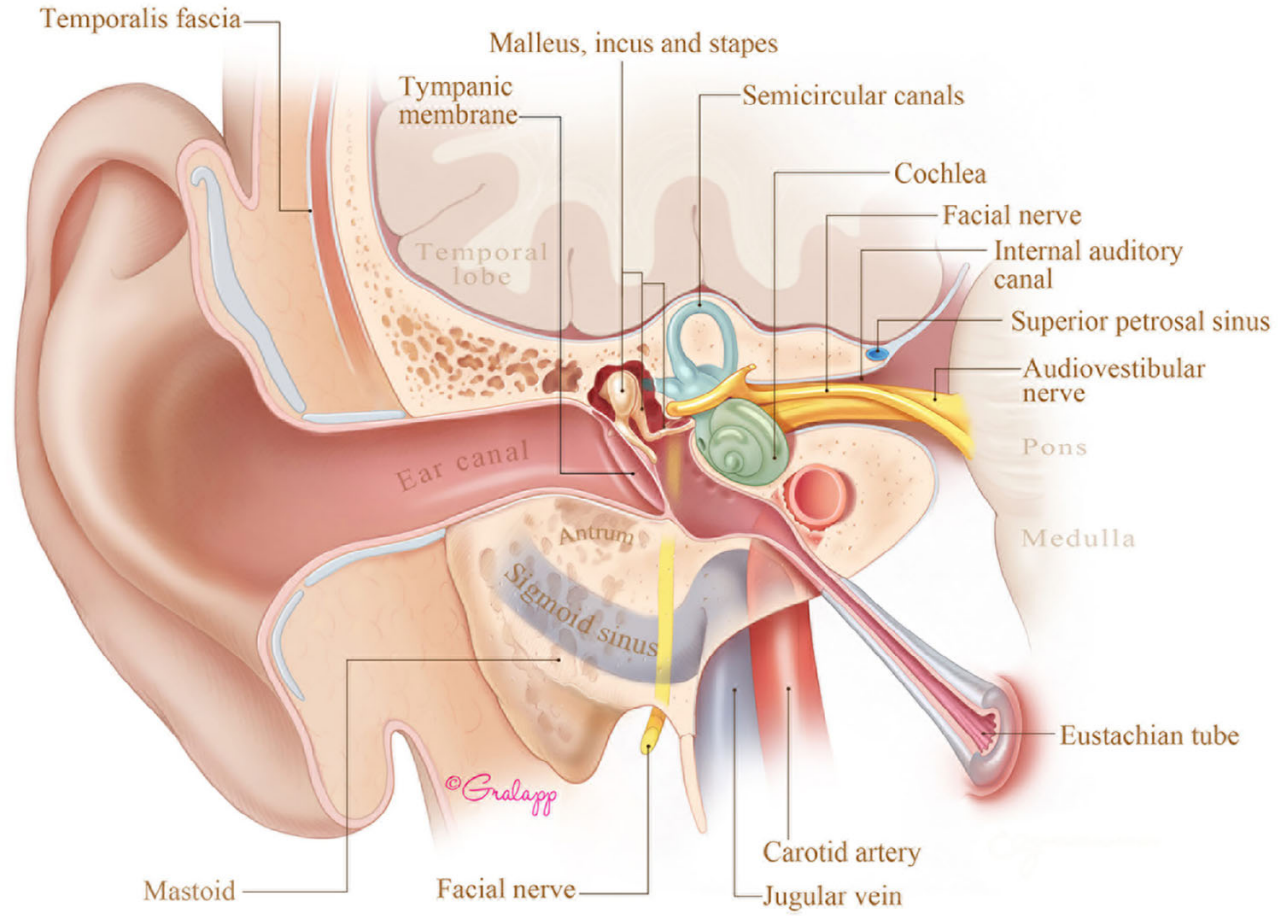
Overview of human inner ear anatomy. The cochlea (green) and vestibular system (blue) are embedded in the temporal bone and innervated by the audiovestibular nerve (orange). Source: Stanford Medicine Otologic Surgery Atlas (https://otosurgeryatlas.stanford.edu/otologic‐surgery‐atlas/surgical‐anatomy‐of‐the‐ear/overview‐of‐temporal‐bone/). Reproduced with permission by the artist, Chris Gralapp.

**Figure 2:**
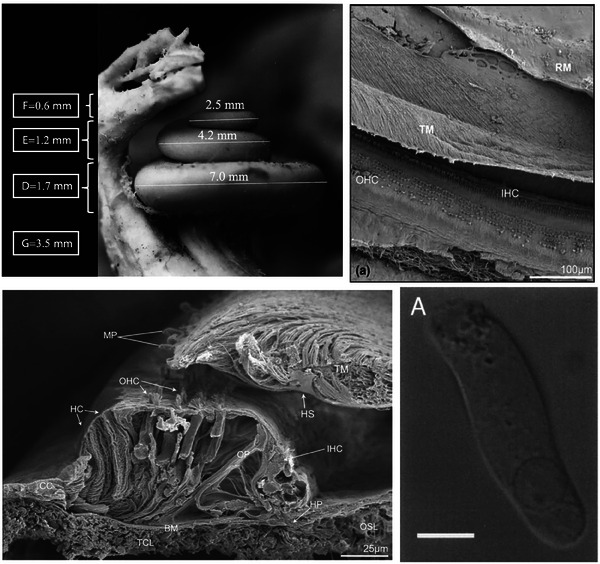
Detailed anatomy of the human cochlea, the organ of Corti, and sensory hair cells. Top left: Corrosion cast of a human cochlea with measurements of the basal, second, and apical turns. The dimensions of the entire human cochlea are ≈7 mm wide and ≈3.5 mm high, and vary across individuals. Reproduced from Erixon et al. with permission.^[^ ^7^ ^]^ Copyright 2009, Wolters Kluwer Health, Inc. The Creative Commons license does not apply to this content (Erixon et al.). Use of the material in any format is prohibited without written permission from the publisher, Wolters Kluwer Health, Inc. Please contact permissions@lww.com for further information. Top right: Scanning electron micrograph (SEM) of the surface of the human organ of Corti, corresponding to the 500 Hz–1 kHz region. There is one row of IHCs and several rows of OHCs. The tectorial membrane (TM) and Reisser's membrane (RM) are also visible. Reproduced from Glueckert et al. with permission.^[^ ^8d^ ^]^ Copyright 2005, Elsevier. Bottom left: SEM cross‐section of the human organ of Corti, corresponding to the 500 Hz region. The tympanic covering layer (TCL) of the basilar membrane (BM), osseous spiral lamina (OSL), TM, IHCs, OHCs, and supporting cells can be seen. CC, Claudius cell; HP, habenula perforata; HS, Hensens stripe; MP marginal pillars; OP, outer pillar. Reproduced from Glueckert et al. with permission.^[^ ^8d^ ^]^ Copyright 2005, Elsevier. Bottom right: OHC isolated from the second turn of the human cochlea. Hair cells are 30–70 µm in length. Scale bar = 10 µm. Reproduced from Oghalai et al. with permission.^[^ ^8b^ ^]^ Copyright 1998, The American Physiological Society.

Across medical disciplines, the holy grail of imaging is the high‐resolution direct visualization of intact, functional tissue without its perturbation. In addition to imaging anatomical structures, detecting dynamic cellular functions can provide deeper insights into human diseases and inform the best course of therapy. However, despite the seminal contributions of otologists Nylén and Holmgren to medical imaging, otolaryngology and otology have not yet benefited from the transformative impact of high‐resolution clinical imaging due to the small size, fragility, structural complexity, and bone‐embedded location of the sensory organs of the human inner ear. The cochlea and peripheral vestibular organs, which respectively detect sound and positional information, are located within the temporal bone, encased by the otic capsule – the densest bone in the human body – presenting challenges for imaging penetration and access (**Figure** **1**).^[^ ^6^ ^]^ Further, the human cochlea is approximately 4–7 mm in diameter,^[^ ^7^ ^]^ while the sensory inner hair cells (IHC) and outer hair cells (OHC) are just ≈10–50 µm in width and 30–70 µm in length,^[^ ^8^ ^]^ respectively, and closely clustered within a fragile and complex sensory epithelium (**Figure** **2**). The anatomy of the spiraling cochlea and looping vestibular system is uniquely intricate, incorporating fluid chambers of differing ionic contents separated by thin, flexible membranes (see Video S1, Supporting Information). Finally, the organs of the inner ear are exquisitely sensitive to physical, biochemical, and vibrational damage, necessitating approaches tailored to a person's unique anatomy to preserve residual hearing and balance functions. Together, these considerations place high demands on an ideal imaging method that could be used in an otologic clinic, either routinely or as a surgical tool. However, emerging imaging modalities have demonstrated promise for overcoming some or all of these challenges.

### 1.1 Importance of Imaging the Human Cochlea

The most common etiologies of hearing loss originate in the inner ear, primarily due to the death or dysfunction of the sensory cells.^[^ ^9^ ^]^ During hearing, sound‐induced vibrations are transmitted from the middle ear to cochlear fluids, resulting in mechanical stimulation of the stereocilia of the IHCs and OHCs, followed by neurotransmitter release by IHCs.^[^ ^10^ ^]^ The hair cells lie within the organ of Corti, the human auditory sensory epithelium, and synapse with spiral ganglion neurons (SGNs) which form the auditory nerve.^[^ ^11^ ^]^ In humans, these sensory cells are present in the cochlea at birth, are not replaced when lost, and have very limited ability for repair.^[^ ^12^ ^]^ Thus, the loss or damage of hair cells and SGNs due to aging (presbycusis),^[^ ^13^ ^]^ loud noise,^[^ ^14^ ^]^ genetic mutations,^[^ ^15^ ^]^ or other reasons (i.e., trauma, ototoxicity, inflammation, or infections)^[^ ^16^ ^]^ all contribute to sensorineural hearing loss (SNHL).

SNHL is a leading sensory deficit worldwide, affecting 1.5 billion people and disabling an estimated 466 million people,^[^ ^17^ ^]^ and an additional 1.1 billion people are at increased risk due to greater longevity and noise exposure.^[^ ^18^ ^]^ As a result, the humanistic burden associated with SNHL is enormous and numerous studies have identified negative impacts on quality of life, productivity, social engagement, mental health, functioning, and safety.^[^ ^19^ ^]^ The economic burden of SNHL is also substantial, with total costs of up to $133 billion annually in the United States (US) alone^[^ ^20^ ^]^ and nearly a trillion dollars annually worldwide.^[^ ^17^ ^]^ Novel therapies to prevent, treat, and ideally reverse SNHL are greatly needed to reduce these burdens, yet these must be tailored to a patient's specific form of hearing loss. The hearing deficit may be mild or profound, present in one ear or both, or may affect certain or all frequencies.^[^ ^21^ ^]^ SNHL may be gradual or sudden^[^ ^22^ ^]^ and can be present from birth or appear in later life.^[^ ^23^ ^]^ Severe or prolonged insults can render the organ of Corti a flat epithelium devoid of sensory cells,^[^ ^24^ ^]^ necessitating the development of cell replacement rather than other therapeutic strategies. Thus, the presentation of SNHL and individual cellular pathology within the cochlea greatly varies from patient to patient.

Understanding the unique aspects of a patient's SNHL and, in particular, the extent and location of sensory cell damage, could inform therapeutic choice now and as new treatments emerge. This need is highlighted by recent breakthroughs in the development of gene therapy for patients with otoferlin gene (*OTOF*) mutations, which depends on the presence of remaining cochlear sensory cells that can be repaired to restore hearing.^[^ ^25^ ^]^ Conventional diagnostic modalities for SNHL during formal audiological testing inform clinicians as to the severity and general type of the hearing deficit,^[^ ^26^ ^]^ but provide no cellular‐level information about the health, function, or remaining density of cochlear sensorineural cells. Therefore, our knowledge of the cellular pathophysiology of SNHL has been largely limited to studies using animal models and specimens from human organ donors. Histological analysis of human specimens is accompanied by inherent artifacts due to decalcification, fixation, staining, and sectioning that can alter morphological features, as well as post‐mortem autolysis.^[^ ^27^ ^]^ Animal models often have limited applicability due to differences in cochlear size, structure, and cellular dynamics versus humans. Compared to mice, the most common animal model for hearing research, the human cochlea is ≈100 times larger by volume,^[^ ^28^ ^]^ the temporal bone is substantially denser,^[^ ^29^ ^]^ and mimicking human‐like forms of hearing loss is challenging.^[^ ^30^ ^]^ This is illustrated by the fact that despite promising evidence in animal models,^[^ ^31^ ^]^ numerous clinical trials of candidate gene and pharmaceutical therapies with the goal of preventing SNHL^[^ ^32^ ^]^ or prompting hair cell regeneration^[^ ^33^ ^]^ have not reported positive results. Consequently, there is no approved treatment indicated for the restoration of human hearing.

The development of high‐resolution imaging of the human cochlea in vivo would be expected to transform the clinical diagnosis of SNHL and catalyze research into novel treatments. In this review, we discuss the benefits and drawbacks of existing medical imaging techniques used to diagnose disorders of the inner ear, as well as those of emerging technologies that may help overcome historical challenges to access, resolution, and functional detail.

## 2 Imaging Techniques for the Human Inner Ear

The quality of medical images is typically assessed in terms of contrast, signal‐to‐noise ratio, and spatial resolution, although the quality ultimately achieved depends on both the intrinsic properties of the imaging system and the acquisition parameters.^[^ ^2^, ^34^ ^]^ For example, the resolution of medical digital images increases with the number of pixels in the field of view, which is a product of the instrumentation and detector sensitivity. Post‐processing of digital images can enable segmentation of tissue, volumetric reconstruction, or filtering to improve quality.^[^ ^35^ ^]^ **Table** **1** summarizes the notable aspects of the imaging modalities discussed below, including their strengths and limitations for otologic applications.

**Table 1:** Parameters of clinical and emerging imaging modalities for the human inner ear.

Imaging Technique	Approach	Specimen	Resolution^a)^	Imaging Time^b)^	Notes
**Current clinical modalities**					
*X‐ray*					
Conventional X‐ray	Non‐invasive, can be used in awake patients to confirm CI placement during surgery	in vivo, whole head or temporal bone	100–175 µm pixel sizes for a digital image and ≈50–100 µm for film	Acquisition of conventional radiographs is within a fraction of a second ranges	Typically static images are used to limit radiation exposure
Conventional CT	Non‐invasive in awake patients; used clinically to evaluate osseous structures of the temporal bone	in vivo, whole head or temporal bone/IAC	0.6 mm axial images, 0.5 mm between images; dual FOV: 180–210 mm. Pixel sizes of ≥200 µm	<10 min, although it can be as fast as seconds	Iodine‐based contrast agents may be used for the evaluation of infection, tumors, or vascular pathology, but not typically for hearing loss
Photon‐counting detector CT	Non‐invasive in awake patients; used clinically for temporal bone imaging among other applications	in vivo, whole head or temporal bone	Pixel sizes 150–200 µm	<10 min, although it can be as fast as seconds	31% lower radiation dose than conventional CT
Cone‐beam CT	Non‐invasive in awake patients; used clinically to evaluate osseous structures of the temporal bone and CI placement	in vivo, whole head or temporal bone	Pixel sizes of 100–200 µm in typical clinical settings	6‐40 s	Can detect anatomical features ≥500 µm in size
*MRI*					
1.5T MRI with gadolinium‐based contrast agent	Non‐invasive in awake patients; used clinically for IAC evaluations	in vivo, whole head or temporal bone/IAC	≈1 to 3 mm	30–60+ min	In routine use for providing contrast for soft tissue (i.e., tumors), but cannot resolve small structures at the cellular level
3T MRI with gadolinium‐based contrast agent	Non‐invasive in awake patients; used clinically for IAC evaluations	in vivo, whole head or temporal bone/IAC	≈0.5 to 2 mm	20–40+ min	Superior signal‐to‐noise and shorter imaging time versus 1.5 T. Prone to banding and susceptibility artifacts with gradient‐recalled echo sequences
*PET*	Non‐invasive in awake patients	in vivo, primarily for detection, staging, or surveillance of head and neck tumors	Typically 4–5 mm voxel size	≈30 min	Can be combined with CT, provides dynamic information with multiple available biologic radiotracers
*Endoscopy*					
Conventional light endoscopy	For inner ear: mastoidectomy with facial recess exposure, exposure of round window niche, and cochleostomy in basal turn	in vivo, during CI implantation or other surgical access to the inner ear	Depends on the instrument, but typically 960×720 or 1920×1080 pixels	Real‐time	Commonly 2.7‐4 mm diameter endoscope, 0‐degree or angled
**Emerging modalities**					
*UHF‐MRI*					
7T MRI	Non‐invasive in awake subjects. 7T is in sparse clinical use; fields >7T are not used in the US for the head	Whole head in vivo or post‐mortem	≈500 µm; ≈0.5×0.5×0.5 mm^3^	≈15 min	Increases in signal‐to‐noise ratio and spatial resolution over 3T at the expense of more degradation by susceptibility artifacts
11.7T MRI	Non‐invasive in awake research subjects (Europe). Ex vivo whole human temporal bone without drilling or decalcification (US)	Whole head in vivo or post‐mortem. Fixed temporal bones from organ donors	≈200 µm, 1 mm slice thickness	4–10 min	Similar concerns as with 7T, noise levels require caution in patients with SNHL
Synchrotron radiation phase‐contrast imaging	Whole temporal bone without drilling or decalcification	Fixed temporal bone from human organ donors or animal models	10 µm	Variable per the sample, up to 2 h	High radiation level beyond clinical safety limits
*OCT*					
Conventional OCT	Invasive; drilled temporal bone or after decalcification	Fixed temporal bone from human organ donors or animal models	10–30 µm	≈10 min	Cellular‐level resolution but subject to speckle noise arising from coherent light detection
µOCT	Invasive; drilled temporal bone or, if paired with endoscopy, probe insertion into RWM	Fixed temporal bone from human organ donors or animal models, or freshly excised cochlea from animal models	1–10 µm	<1 to 10 min	Sub‐cellular‐level resolution; can be paired with flexible optics, capable of detecting structural information; no fluorophore required
DµOCT	Invasive; drilled temporal bone or, if paired with endoscopy, probe insertion into RWM	Freshly excised cochlea from animal models	1–10 µm	<1 to 10 min	Sub‐cellular resolution; can be paired with flexible optics, capable of structural and metabolic imaging; no fluorophore required
***Other***					
Ultrasound	Drilled temporal bone or after decalcification	Fixed temporal bone from human organ donors or animal models	Axial resolution 0.05–0.5 mm depending on frequency	Variable, 20 min up to 3 hours	Low resolution of inner ear structures; uses a large (e.g., 8 mm^[^ ^155^ ^]^) probe
Fluorescence microendoscopy	Invasive; drilled temporal bone and otic capsule in vivo or ex vivo	Fixed temporal bone from human organ donors or animal models	≈1.3–3 µm	5–10 min+	Sub‐cellular resolution but rigid optics (350–1,000 µm diameter) require a cochleostomy or an imaging window; needs a fluorophore and is prone to photobleaching

Abbreviations: CI, cochlear implant; CT, computed tomography; DµOCT, dynamic micro‐optical coherence tomography; FOV, field of view; IAC, internal auditory canal; MRI, magnetic resonance imaging; OCT, optical coherence tomography; µOCT, micro‐optical coherence tomography; PET, positron emission tomography; RWM, round window membrane; SNHL, sensorineural hearing loss; T, Tesla; UHF, ultra‐high field; US, United States;

^a)^ Resolution depends on many experimental conditions, including the specimen's characteristics and size, imaging time and parameters, the optics and equipment used, etc. Therefore, the ranges provided should be considered as a general guide and may not be applicable to all imaging sessions;

^b)^ Exclusive of time for surgical approach or other preparation, the total time needed for an imaging session may vary with the imaging parameters.

### 2.1 Imaging Techniques in Current Clinical Use

#### 2.1.1 Conventional X‐Ray

X‐rays are a form of high‐energy electromagnetic radiation that can penetrate living tissue to non‐invasively identify bone structures via projectional radiography.^[^ ^36^ ^]^ The high calcium content of bone, which efficiently absorbs X‐rays, helps it appear starkly visible against tissue or fluids on radiographs. Similarly, X‐rays can be useful for identifying calcifications or foreign objects in tissues, or to guide the insertion of medical devices. On the other hand, body structures low in calcium poorly absorb X‐rays and appear as hazy shadows. As X‐ray uses ionizing radiation to generate images, which is potentially carcinogenic, precautions must be taken to protect the patient from unnecessary and harmful exposure.^[^ ^37^ ^]^ For this reason, conventional X‐ray radiographs are typically static images with a goal of short exposure time.

Due to the distinct bony elements of the temporal bone and middle ear, X‐ray was used in the early half of the 20^th^ century to diagnose otitis media or mastoiditis, infections of the middle ear and mastoid bone, respectively.^[^ ^38^ ^]^ Specialized oblique projections that highlight the temporal bone and bony labyrinth, such as the Stenvers and Pöschl views, were initially developed with traditional X‐ray and are still in modified use today. The Stenvers view is often used to visualize the petrous temporal bone and aid in the identification of cerebello‐pontine‐angle tumors,^[^ ^39^ ^]^ while the Pöschl view is a coronal reconstruction useful for visualization of the semicircular canals.^[^ ^40^ ^]^ In current otology practice, X‐ray may be used to detect temporal bone disease^[^ ^41^ ^]^ or confirm cochlear implant (CI) electrode placement via an intraoperative post‐insertion skull radiograph.^[^ ^42^ ^]^

**Figure 3:**
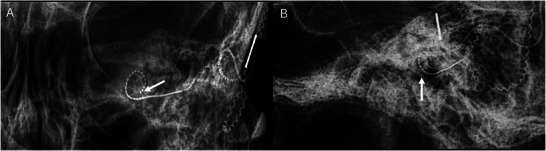
Radiographs of the temporal bone and inner ear following cochlear implantation. Radiographs in the anteroposterior (A) and lateral (B) views demonstrate the electrode array positioned within the cochlea (arrow).

The spatial resolution of an X‐ray radiograph ranges from 100–175 µm (i.e., pixel size) for a digital image and ≈50‐100 µm for film.^[^ ^36^ ^]^ Thus, the resolution is sufficient to discern the gross anatomical location of the CI electrode in the cochlea (**Figure** **3**), but smaller anatomy or non‐calcium‐ or metal‐containing features are not detected. Although the clinical applications of traditional X‐ray are limited, X‐ray has enabled more sophisticated imaging modalities that are in widespread clinical use such as computed tomography (CT).

#### 2.1.2 Computed Tomography

CT is a non‐invasive imaging technique that utilizes a rotating X‐ray source to emit X‐rays at multiple angles around the body. These X‐rays are subsequently detected by opposing detectors and the acquired data are processed to generate a series of 2D images.^[^ ^43^ ^]^ The intensity of the X‐rays that reach the detectors varies depending on the density of the tissues they pass through and determines the contrast of the image. The 2D images are compiled into 3D greyscale “stacks” (i.e., a tomogram) via a reconstruction algorithm to reveal internal body structures, including the skull and temporal bone (Video S2, Supporting Information). Modern CT systems operate using a radiation dose that poses little risk to human health,^[^ ^44^ ^]^ enabling repeated or longitudinal scans to monitor structural changes like tumor growth or response to therapy.^[^ ^45^ ^]^ CT images can be analyzed from any direction through multi‐planar reformats, and various post‐processing techniques can be used to enhance the structures of interest, including color‐coding to highlight specific details or anatomy. The spatial resolution of computed tomograms depends on the detector focal spot and voxel size, with smaller voxels requiring a smaller area to be imaged and field of view but yielding higher resolution. Conventional CT scanners commonly used in medical imaging typically have voxel sizes of ≥200 µm.^[^ ^43^ ^]^ Stabilizing the specimen/patient is important during CT scanning to avoid motion artifact, which can result in image blurring and degradation.

In the otology clinic, CT is commonly used to image the temporal bone to detect inner ear pathology, including bony anatomical conditions or malformations (i.e., otosclerosis or congenital defects).^[^ ^46^ ^]^ Pöschl and Stenvers views are used to optimize visualization of the superior semicircular canal in CT, particularly for suspected dehiscence. Oriented parallel (Pöschl) and perpendicular (Stenvers) to the superior semicircular canal, these planes enhance detection of bony defects along the arcuate eminence compared to standard axial or coronal planes.^[^ ^47^ ^]^ Less commonly, CT is used to evaluate conditions affecting the auditory nerve or central auditory pathway, especially in subjects unable to undergo MRI. Additionally, CT scans may be performed before CI implantation to inform the surgeon as to the gross anatomical characteristics of the candidate's cochlea, essential for determining suitability and the ideal surgical approach.^[^ ^48^ ^]^ While CT is preferred, modified Stenvers radiographs can also be useful for assessing CI positioning.^[^ ^42^, ^49^ ^]^

**Figure 4:**
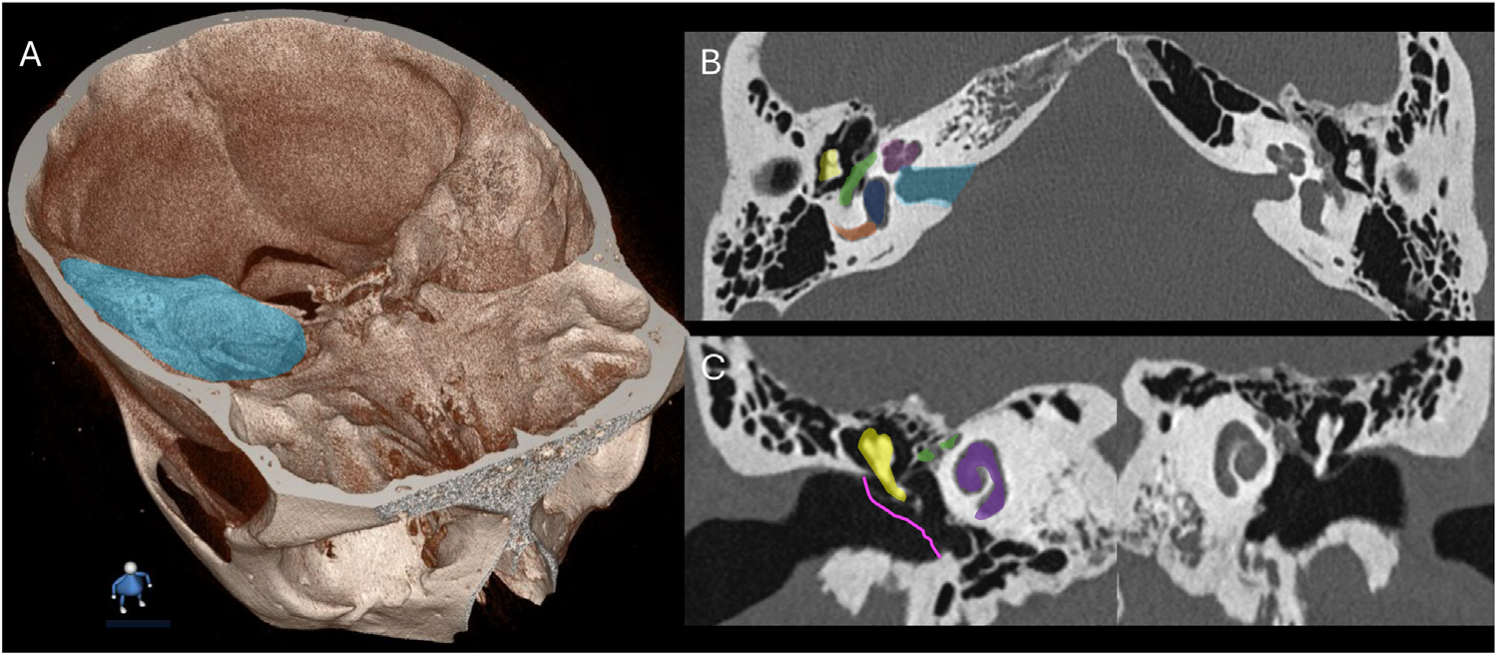
3D computed tomography (CT) of the human temporal bone and inner ear. A) CT of a human skull with the temporal bone highlighted in blue. Corresponding B) axial and C) coronal CT images demonstrating normal temporal bone structures, color‐coded on the left side (patient's right side) for reference to the opposite side. Purple, cochlea. Yellow, ossicles. Green, facial nerve. Light blue, internal auditory canal. Dark blue, vestibule. Orange, posterior semicircular canal. Pink, tympanic membrane.

**Figure 5:**
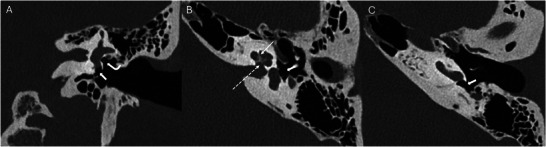
Photon‐counting detector computed tomography (PCD CT) images of the spiral structures within the human cochlea. Normal left temporal bone anatomic structures on PCD CT. A) Coronal PCD CT image demonstrating the position of the oval window (curved arrow) and round window (straight arrow). B) Axial PCD CT image at the level of the oval window (thick arrow) with insertion of the stapes crus. The modiolus (dashed arrow) and spiral lamina of the cochlear (thin solid arrow) can also be seen. C) At an inferior axial CT level, the round window is seen along the basal turn of the cochlea (arrow).

**Figure 6:**
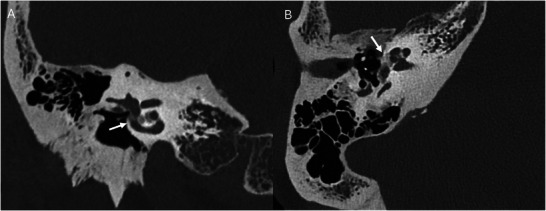
Photon‐counting detector computed tomography (PCD CT) images of otospongiosis. Coronal (A) and axial (B) PCD CT images showing lucency in the otic capsule involving the right fissula ante fenestram, oval window, cochlea, vestibule, and facial nerve canal compatible with otospongiosis (arrow).

**Figure 7:**
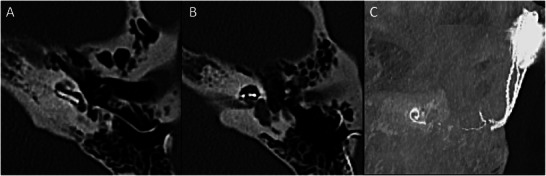
Cone‐beam computed tomography (CBCT) images of a left cochlear implant in situ. Axial CBCT images demonstrate the cochlear lead entering the mastoid bowl and round window to the basal turn (A) and middle turn (B) of the cochlea. (C) A 3D maximum‐intensity projection of the CBCT images demonstrates the position of the cochlear implant on a coronal oblique view.

Temporal bone CT enables the acquisition of axial images, which can then be reconstructed in any plane (**Figure** **4**). A new technology called photon‐counting detector CT (PCD CT) directly records individual X‐ray photons as electric signals, permitting small detector pixels (≈150 µm) at a reduced radiation dose (by 31%) and with fewer artifacts than conventional CT.^[^ ^50^ ^]^ PCD CT is being rapidly adopted for temporal bone imaging given these benefits,^[^ ^51^ ^]^ and is useful for visualizing small anatomical structures of the inner ear (**Figure** **5**) and pathology such as otospongiosis (i.e., formation of soft bone that can lead to otosclerosis and hearing loss) (**Figure** **6**). However, PCD CT cannot delineate the cells of the cochlea.^[^ ^52^ ^]^ Cone‐beam computed tomography (CBCT) is a variation of CT that uses a cone‐shaped X‐ray beam to create images of the head and neck.^[^ ^53^ ^]^ CBCT provides several advantages over multidetector CT (MDCT) for temporal bone imaging, including superior spatial resolution, reduced metallic artifacts, lower radiation dose, and faster imaging speed.^[^ ^54^ ^]^ This allows for precise delineation of gross cochlear anatomy—specifically, the cochlear walls, modiolus, and osseous spiral lamina—to optimize surgical planning, especially for cochlear implantation (**Figure** **7**). Furthermore, CBCT has demonstrated superior post‐CI visualization and image quality compared to MDCT.^[^ ^55^ ^]^ However, clinical CBCT is limited to the detection of anatomical features of approximately ≥500 µm in size^[^ ^56^ ^]^ and is therefore unable to provide cellular‐level resolution of the cochlea.

#### 2.1.3 Positron Emission Tomography (PET)

PET is a functional imaging method that uses positrons and radiotracers (delivered intravenously) to visualize and measure changes in dynamic body functions, including metabolism, blood flow, biochemical shifts, and absorption.^[^ ^57^ ^]^ The choice of radioisotope depends on the target being imaged, such as Oxygen‐15 for blood flow or [^18^F]‐fluoro‐2‐deoxy‐D‐glucose (18F‐FDG) and 68‐gallium dotatate for tumor detection, and the options are constantly expanding.^[^ ^58^ ^]^ Decay of the radioisotope results in the emission of positrons that interact with electrons to emit opposing gamma rays.^[^ ^57a^ ^]^ The PET scanner then detects the gamma rays and constructs a 3D image.

**Figure 8:**
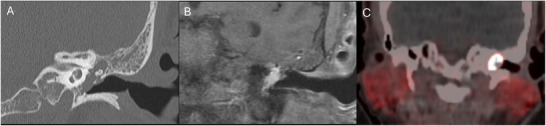
Multi‐modal imaging of a glomus tympanicum with positron emission tomography/computed tomography (PET/CT) and magnetic resonance imaging (MRI). Imaging of a left glomus tympanicum, a benign paraganglioma of the middle ear, which is highly vascularized. The mass completely opacifies the middle ear cavity on coronal CT (A), with avid enhancement on MR (B) and 68‐gallium dotatate hypermetabolism on PET/CT (C).

The spatial resolution of conventional PET images depends on the radioisotope but is typically 4–5 mm voxel size, a benchmark that has remained stable over time due to the minimal changes that can be made to the physical effects affecting resolution in PET cameras.^[^ ^59^ ^]^ Therefore, PET is typically used for the detection of larger tissue structures (i.e., metastases) or changes in tissue or tumor blood flow and/or metabolism over time. PET is a flexible imaging method given the range of available radioisotopes, and that PET and CT scanners can be combined within a single machine (PET/CT) to provide multi‐dimensional data.^[^ ^60^ ^]^ PET or PET/CT are commonly used to aid in the diagnosis and staging of head and neck tumors with high sensitivity (see glomus tympanicum imaging in **Figure** **8**),^[^ ^61^ ^]^ and are employed to evaluate auditory cortical activity following CI implantation.^[^ ^62^ ^]^ However, despite the flexibility of this modality, the resolution of PET precludes the capture of dynamic processes occurring at the cellular level within the cochlea or vestibular system.

#### 2.1.4 Magnetic Resonance Imaging

MRI is a non‐invasive imaging technique that uses strong magnets and low‐energy radiofrequency signals to generate images of anatomical structures via nuclear magnetic resonance (reviewed in detail by Grover et al.^[^ ^63^ ^]^ and Liang et al.^[^ ^64^ ^]^). While MRI captures a “snapshot” image, functional MRI (fMRI) records dynamic metabolic processes over time, such as changes in neural activity (i.e., using blood oxygenation level dependent [BOLD] imaging).^[^ ^65^ ^]^ Compared to CT, MRI provides superior images of soft tissue structures as the signal best maps to water and fat in the body, which contain abundant hydrogen atoms that emit a detectable signal.^[^ ^66^ ^]^ Thus, MRI of anatomical structures or blood flow does not necessarily require contrast agents (e.g., gadolinium), although they may be used to enhance specific anatomical structures or when conducting fMRI.

The resolution of MRI images is defined by the number of pixels in a specified field of view in two dimensions and the size of the imaging voxels in 3 dimensions (3D rectangles). Thus, the size of the voxel and the resolution depend on parameters such as matrix size (i.e., the number of frequency encoding steps), field of view, imaging slice thickness, and the magnet field strength.^[^ ^63^ ^]^ Increases to the field strength commensurately increase the spatial and temporal resolution of the images, but also the signal‐to‐noise ratio, acoustic noise, and specific absorption rate (i.e., the energy absorbed by the body).^[^ ^67^ ^]^ MRI systems commonly used in clinical practice have a field strength of 1.5 to 3 Tesla (T).^[^ ^63^, ^68^ ^]^ The typical spatial resolutions range from approximately 1 to 3 mm for 1.5 T MRI and 0.5 to 2 mm for 3T MRI, depending on the protocol, sequence parameters, and the anatomy being imaged.^[^ ^69^ ^]^ Clinical systems up to 7T, termed ultra‐high‐field MRI (UHF‐MRI), have been recently cleared by the US Food and Drug Administration (FDA) but are not in common use;^[^ ^70^ ^]^ therefore, UHF‐MRI is discussed in the next section.

**Figure 9:**
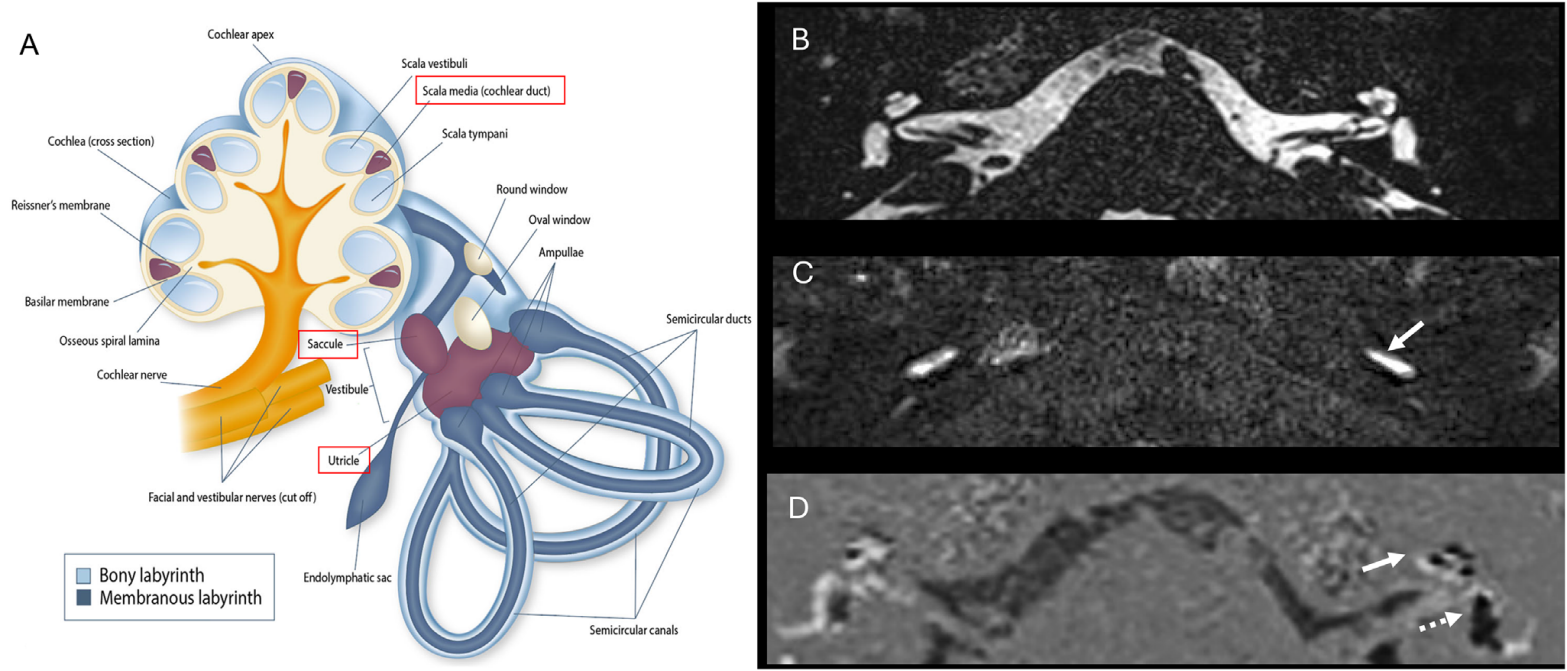
Magnetic resonance imaging (MRI) of vestibular and cochlear endolymphatic hydrops. Hydrops is a pathological increase of fluid in the cochlear duct (i.e., endolymph) and/or membranous labyrinth of the vestibular system (purple shading in A). B–D) Axial MRI of a patient with left greater than right vestibular and cochlear endolymphatic hydrops with dilatation of the cochlear duct as well as the utricle and saccule, resulting in confluence of these vestibular structures. Conventional cisternogram (B) appears normal and cannot discriminate between the perilymphatic and endolymphatic spaces. A heavily T2‐weighted 3D‐FLAIR axial image (C) indicates there is asymmetric enhancement of the left (arrow in C) greater than the right basal turn of the cochlea, indicative of blood‐labyrinthine barrier contrast leakage. Subtraction images from heavily T2‐weighted 3D‐FLAIR axial images demonstrate left greater than right cochlear (solid arrow in D) and vestibular (dashed arrow in D) endolymphatic hydrops. Diagram in (A) adapted from de Pont et al.^[^ ^78^ ^]^ under a Creative Commons Attribution 4.0 International License (http://creativecommons.org/licenses/by/4.0/). Copyright 2020, Springer Nature.

In otology, gadolinium contrast‐enhanced T1 MRI is commonly used for evaluation of asymmetric hearing loss via imaging the internal auditory canal or cerebellopontine angle (i.e., for detection of vestibular schwannoma or related tumors).^[^ ^71^ ^]^ Additionally, MRI, typically with contrast agents, may be used during patient screening for a CI^[^ ^72^ ^]^ or to aid in the identification of pathologies such as cholesteatoma or endolymphatic hydrops (i.e., Meniere's disease), the latter of which requires a four‐hour delay in imaging after intravenous contrast administration for optimal discrimination of the endolymphatic and perilymphatic space (**Figure** **9**).^[^ ^73^ ^]^ T2‐weighted steady‐state free precession sequences, such as constructive interference in steady state (CISS) and fast imaging employing steady‐state acquisition (FIESTA), may be used to image the skull base due to the high natural contrast between cerebrospinal fluid (CSF) and surrounding tissue (i.e., cranial nerves).^[^ ^74^ ^]^ CISS or FIESTA offer improved CSF hyperintensity, signal‐to‐noise ratio, contrast‐to‐noise ratio, and spatial resolution compared to conventional MRI.^[^ ^75^ ^]^ As a result, CISS/FIESTA have become the sequences of choice for CSF‐cisternography and are commonly used to assess for cholesteatoma or vestibular schwannoma in cases of asymmetric SNHL.^[^ ^76^ ^]^ However, the microstructures of the inner ear cannot be clearly visualized with CISS/FIESTA due to insufficient resolution and banding artifacts from variations in magnetic fields.^[^ ^77^ ^]^

**Figure 10:**
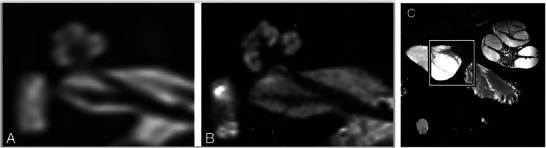
3T (A), 7T (B), and 11.7T MRI of the human cochlea. Axial cross‐section of a human inner ear and cochlea in vivo using 3T (A) and 7T (B) MRI. Reproduced from van der Jagt et al. with permission.^[^ ^82^ ^]^ Copyright 2015, American Journal of Neuroradiology; permission conveyed through Copyright Clearance Center, Inc. C) Membranous anatomy of human cochlea from organ donors and utricular macula (boxed area) ex vivo using 11.7T MRI. Reproduced from Thylur et al. with permission.^[^ ^83^ ^]^ Copyright 2017, Wolters Kluwer Health, Inc. The Creative Commons license does not apply to this content (Thylur et al). Use of the material in any format is prohibited without written permission from the publisher, Wolters Kluwer Health, Inc. Please contact permissions@lww.com for further information.

However, 1.5 and 3T MRI cannot achieve cellular‐level resolution of the inner ear and are limited to the detection of fluid signal within bony or membranous labyrinth and the gross anatomy of the auditory nerve (**Figure** **10**a).^[^ ^71^, ^72^, ^79^ ^]^ Videos S3 and S4 demonstrate T2 MRI‐weighted cine images of normal inner ear structures in axial and coronal planes, respectively. The benefits of MRI include its non‐invasiveness, routine use, instrumentation across clinics/hospitals, and lack of ionizing radiation in an outpatient setting. Drawbacks include noise level, limitations to resolution, and the exclusion of patients with contrast agent allergy or certain implants or metal devices, particularly with 3T MRI.^[^ ^80^ ^]^ Additionally, temporary hearing threshold shifts have been reported with MRI even at lower (1.5 T) field strengths,^[^ ^81^ ^]^ requiring cautious use in patients with SNHL.

#### 2.1.5 Endoscopy

Endoscopes direct light from an external source through a series of prisms/lenses or fiberoptic bundles to capture an image, which is transmitted back to the viewer or to a computer for processing.^[^ ^84^ ^]^ Endoscopy provides the ability to directly visualize the lumens and tissues deep within body cavities with minimal artifact and is suitable for pairing with multiple imaging methods. Additionally, endoscopic instrumentation can allow precise manual manipulation by the physician as well as exceptional resolution, contrast, and magnification. The close proximity of the endoscopy lens to the tissue of interest can provide enhanced depth of field, while the application of prisms can permit various viewing angles.

**Figure 11:**
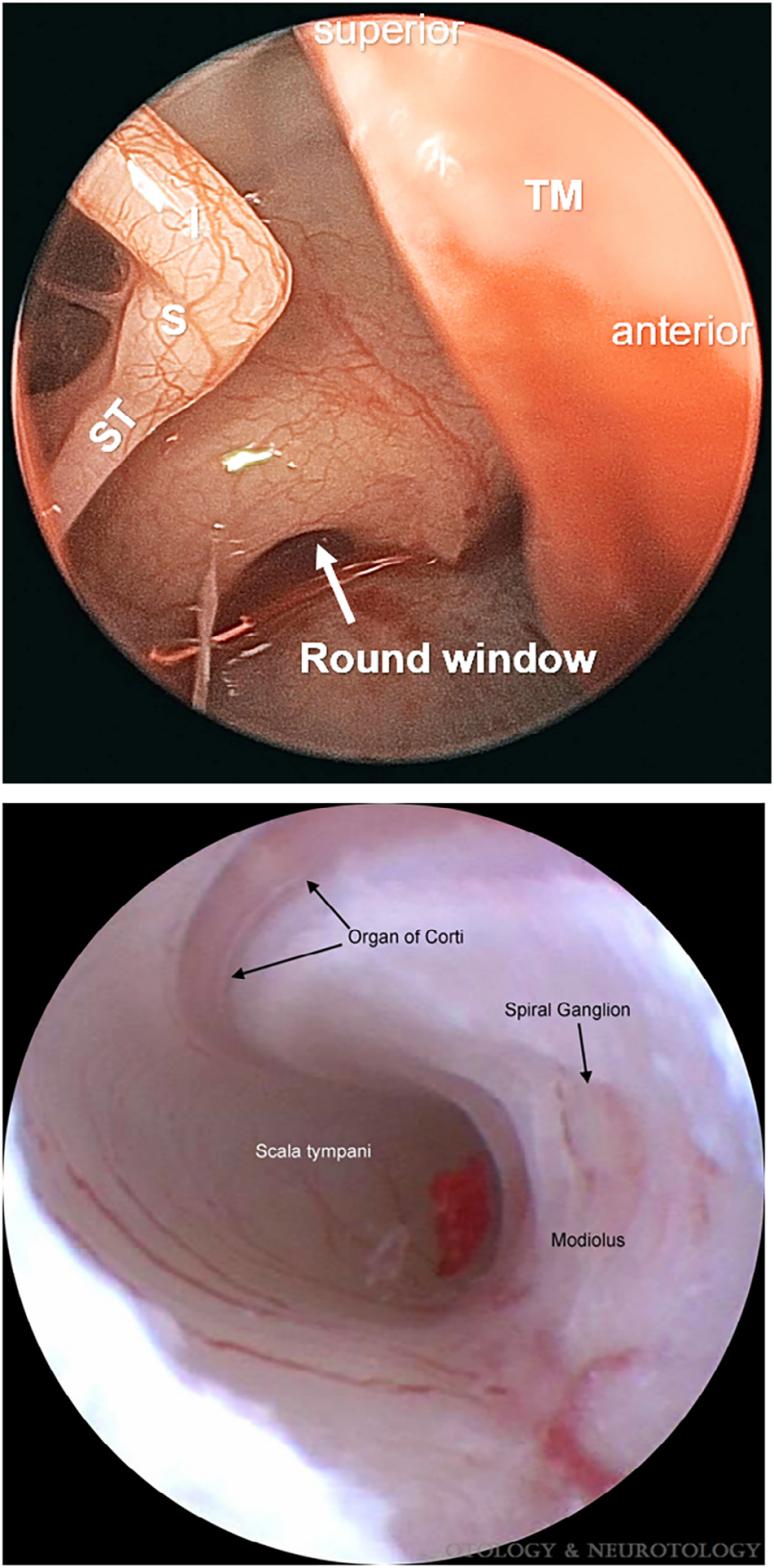
Endoscopy of the middle ear and round window (top) and the scala tympani (bottom). (Top image) Light endoscopy is used during surgical access to the middle ear. The stapes (S) with stapedial tendon (ST), incus (I), and tympanic membrane (TM) reflected anteriorly are seen (0° endoscope). (Bottom image) Additionally, light endoscopy has been used to visualize the scala tympani prior to CI electrode placement; in this case, the middle ear and mastoid were filled with isotonic saline. Interior structures of the cochlea that can be visualized with light endoscopy include the basal turn of the scala tympani, the modiolus, spiral ganglion, microvasculature, and organ of Corti. Image in bottom panel reproduced from Chole et al. with permission.^[^ ^87^ ^]^ Copyright 2015, Wolters Kluwer Health, Inc. The Creative Commons license does not apply to this content (Chole et al.). Use of this material in any format is prohibited without written permission from the publisher, Wolters Kluwer Health, Inc.

A wide variety of straight or flexible endoscopes are routinely used in the otology clinic to image the ear canal, the middle ear (top panel in **Figure** **11**) and mastoid antrum (during procedures such as tympanoplasty or microdissection of cholesteatoma), and inner ear (during placement of a CI electrode via the round window).^[^ ^85^ ^]^ Rigid light‐based endoscopes used in the human inner ear (e.g., Leica) are approximately 11–18 cm in length, 2.7–4 mm in diameter, may have 0° or angled (30° or 45°) views, and are connected to a high‐resolution digital camera (e.g., Karl Stortz [1920×1080 pixels]).^[^ ^85^, ^86^ ^]^ Due to the coiling shape of the cochlea and the large diameter of clinically available rigid endoscopes, they are destructive when entering the cochlea and are therefore unsuitable when hearing preservation is a goal. Chole et al.^[^ ^87^ ^]^ used light endoscopy to visualize the scala tympani of a two‐year‐old undergoing CI surgery (bottom panel in Figure 11), although the imaged area was constantly irrigated with isotonic saline due to the inability to image through perilymph, and to clear away blood and mitigate thermal effects of the endoscope. Miniaturization of micro‐electro‐mechanical systems and use of fiberoptic bundles have enabled the development of smaller (0.8–1.2 mm), flexible endoscopes that are less invasive and have been used in skull base surgery (i.e., resection of vestibular schwannoma).^[^ ^88^ ^]^ The resolution and signal‐to‐noise ratio of fiberoptic endoscopes depend on the size, number, and alignment fidelity of the bundles, with each fiber relaying one pixel.^[^ ^89^ ^]^

### 2.2 Emerging Imaging Modalities

Despite the demonstrated utility of the current clinical imaging methods for detecting larger anatomical features like tumors or bone malformations, none possess sufficient resolution for in vivo cellular‐level imaging of the structure or dynamic metabolism of the sensorineural cells of the human cochlea. This has prompted investigations into new imaging modalities or the improvement of current techniques, which are summarized below and in Table 1.

#### 2.2.1 Ultra‐High‐Field MRI

As conventional 3T MRI cannot resolve the microanatomy of the inner ear (Figure 10A), MR systems with stronger magnetic fields have been tested for this purpose. UHF‐MRI describes forms of MRI ≥7T, currently above that which is commonly used clinically. In 2017, the Magnetom Terra MRI (Siemens Medical Solutions Inc.) was the first and currently remains the only 7T system cleared for clinical use by the FDA.^[^ ^70^, ^90^ ^]^ The reported resolution of the Magnetom Terra 7T MRI is 0.2 mm in‐plane with 1 mm slice thickness.^[^ ^91^ ^]^ Larger features of the human inner ear, such as the osseous spiral lamina and the labyrinthine artery, can be resolved at 7T in vivo (Figure 10B), but smaller structures including the organ of Corti still cannot be distinguished due to the spatial resolution of ≈300 µm at this field strength.^[^ ^82^, ^92^ ^]^ Increasing the field strength to 11.7T has allowed visualization of the basilar membrane, Reissner's membrane, and the scala media of the cochlea and gross anatomical features of the vestibular system in extracted human temporal bones (Figure 10C).^[^ ^83^ ^]^ However, the resolution remains insufficient for detecting structures <200 µm in the inner ear or for cellular‐level detail. Additional drawbacks of UHF‐MRI include their considerable expense relative to lower‐field strength MRI systems as well as heightened safety concerns, including imaging‐induced vertigo, due to the stronger magnetic fields.^[^ ^93^ ^]^

#### 2.2.2 Ultra‐High Resolution CT

Micro‐CT and nano‐CT are advanced versions of CT that achieve micrometer (voxel sizes ≥0.1 µm) and nanometer resolutions (down to voxel sizes ≈10 nm), respectively, in 3D tomograms.^[^ ^43^ ^]^ The modalities may be based on X‐ray tubes or synchrotron sources and, in medical research applications, have been used to investigate bone microarchitecture, vascularization, tumor growth, and organ development.^[^ ^94^ ^]^ Micro‐CT and nano‐CT are used in fixed specimens ex vivo (i.e., for research or clinical pathology)^[^ ^95^ ^]^ and in small animal models in vivo.^[^ ^96^ ^]^

**Figure 12:**
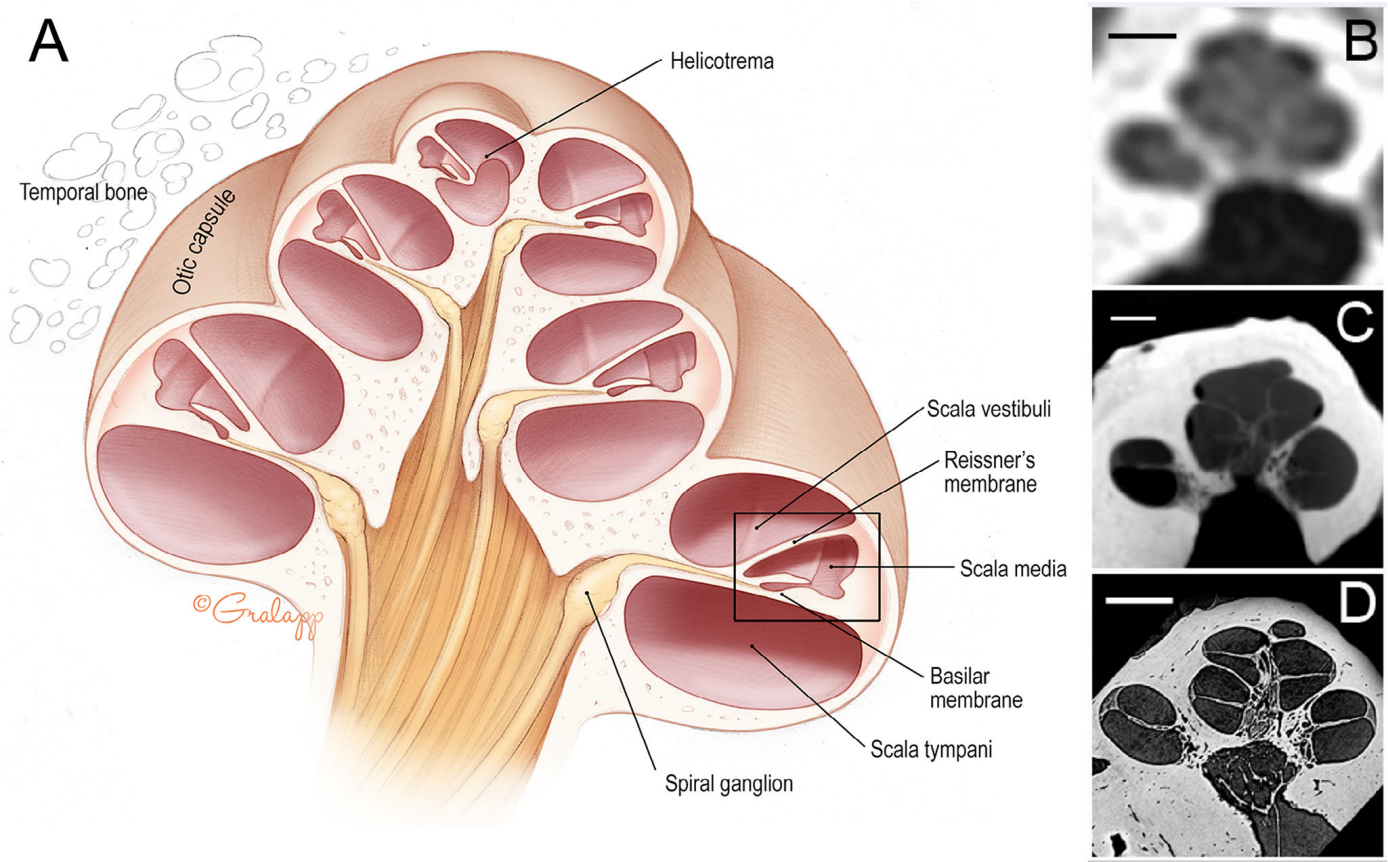
The human cochlea visualized with conventional computed tomography (CT), micro‐CT, and synchrotron radiation phase‐contrast imaging (SR‐PCI). A diagram illustrating the anatomy of the human cochlea with a mid‐modiolar view (A). Conventional CT (B), micro‐CT (C), and SR‐PCI (D) images of virtual mid‐modiolar sections of human cochlea (organ donors) ex vivo. All scales = 2 mm. Illustration in (A) reproduced with permission by the artist, Chris Gralapp. Panels B‐D are reproduced from Iyer et al.^[^ ^97^ ^]^ under the terms of the OSA Open Access Publishing Agreement. Copyright Copyright 2018, Optical Society of America.

Several studies have demonstrated the ability of micro‐CT or nano‐CT to resolve the ossicles and cochlear partitions (i.e., the scalae and basilar membrane) (**Figure** **12**C) or create 3D volumetric reconstructions from fixed specimens from animals or humans.^[^ ^97^ ^]^ Among these, one study visualized Reissner's membrane and SGN bundles, but not individual SGN fibers or hair cells, in human fetal cochlea samples, achieving spatial and axial resolution of 7 and 40 microns, respectively.^[^ ^98^ ^]^ Additionally, micro‐CT has been used to create 3D reconstructions of the human cochlea from histological slides, although the axial resolution of these samples was ≈6 mm due to the sectioning thickness.^[^ ^99^ ^]^ Micro‐CT has also been used to assess trauma to the cochlea resulting from CI electrode implantation using drilled human temporal bones from organ donors.^[^ ^100^ ^]^ The soft tissues of the inner ear were dehydrated and embedded in glycol methyl methacrylate resin prior to imaging, achieving a resolution of 11.4 microns, although contrast was poor and included substantial artefacts from the CI electrode. Additionally, a study by Glueckert et al. used micro‐CT to visualize neurons and hair cells within decalcified, fixed cochleae from mice and humans, achieving 3.0 µm voxel size in humans.^[^ ^97f^ ^]^ However, extraction of the cochlea is required for this method of processing, making it unsuitable for clinical use in vivo. Similarly, while micro‐CT is helpful for identifying the position of the electrode array within the cochlea, histological analysis is required to determine the extent of the damage at the cellular level.

Barriers to clinical translation include the substantial radiation dose needed to achieve high resolution (i.e., 10–50 centigray per scan^[^ ^101^ ^]^), longer scanning time compared to conventional CT, tendency for motion artifacts (i.e., the sample/subject must remain very still to avoid blurring), and need for contrast‐enhancing agents for applications involving soft tissues due to low X‐ray absorption.^[^ ^94^ ^]^ Generating contrast in the organ of Corti in animal models in vivo has remained challenging despite various approaches (e.g., gold nanoparticles and liposomal iodine^[^ ^102^ ^]^). Further, while a conventional CT tomogram can be captured within seconds, the scanning time for micro‐CT is over 30 min and is even longer for nano‐CT.^[^ ^98^ ^]^

#### 2.2.3 Synchrotron Radiation Phase‐contrast Imaging (SR‐PCI)

SR‐PCI is an X‐ray‐based phase contrast tomography technique (a detailed review is by Endrizzi et al.^[^ ^103^ ^]^). Synchrotron radiation imaging of the cochlea, as first described by Vogel et al. in fixed human inner ears, achieved a 10 µm spatial resolution, but the contrast was too poor to delineate the borders of soft tissue structures.^[^ ^97b^ ^]^ To overcome this issue, staining agents (i.e., 1% OsO_4_ in 0.05 M cacodylate buffer) in excised fixed temporal bones and cochleae^[^ ^104^ ^]^ or phase contrast in unstained, intact fixed samples have been used to visualize the organ of Corti and SGN fiber bundles.^[^ ^97^, ^105^ ^]^ SR‐PCI has also been recently used to construct a 3D tonotopic map of the cochlea using fixed whole human temporal bones.^[^ ^106^ ^]^ Additionally, SR‐PCI has been used to measure the morphology of the scala tympani from organ donors who did or did not receive cochlear implants.^[^ ^107^ ^]^ This method approaches the level of detail and contrast available with current histology techniques (Figure 12D), but the levels of radiation energy and length of scanning time needed for sufficient resolution are too high for clinical translation and in vivo use in humans.^[^ ^108^ ^]^

#### 2.2.4 Fluorescence Microscopy and Microendoscopy

Blood supply to the cochlea is partially isolated from that of the rest of the body due to the stria vascularis, which serves as a blood‐cochlea (or blood‐labyrinth) barrier. The stria vascularis selectively passes ions, nutrients, and blood contents to the cochlea and establishes the endocochlear potential of the fluids bathing the IHCs and OHCs necessary for hearing (i.e., endolymph and perilymph).^[^ ^109^ ^]^ While this barrier is less tight than previously believed,^[^ ^110^ ^]^ it can nonetheless restrict cochlear access of intravenous fluorophores or radiotracers that are commonly used to create contrast in medical imaging, instead requiring their application directly to intracochlear fluids.^[^ ^111^ ^]^ However, fluorescent labeling agents that can be internalized by hair cells (i.e., styryl dyes like FM‐143) when applied to intracochlear fluids are often cytotoxic in vivo.^[^ ^112^ ^]^ Therefore, label‐free fluorescence microscopy has been investigated for imaging the cells of the inner ear, focusing on biological sources like autofluorescence or second and third‐harmonic generation microscopy of actin‐tubulin.

Two‐photon (2p) fluorescence microscopy has been used to image cytoarchitecture in fixed, unstained human cochlea samples (from organ donors) ex vivo.^[^ ^113^ ^]^ However, the endogenous fluorescent signal may be, at least in part, an artifact of formaldehyde fixation, potentially due to second harmonic generation following actin polymerization.^[^ ^114^ ^]^ On the other hand, when imaging freshly harvested, unfixed mouse cochleae through the intact round window using 2p excitation fluorescence (TPEF), the intensity of the fluorescence in hair cells was even brighter than in the fixed specimens, suggesting that the observed TPEF signal in the fixed samples was primarily endogenous.^[^ ^115^ ^]^ Relevantly, flavin adenine dinucleotide (FAD) is a major endogenous fluorophore in the inner ear, and its peak of emission (≈520 nm) coincided with the recorded TPEF signal.^[^ ^116^ ^]^ Moreover, the sensory epithelium of the inner ear has one of the highest known tissue concentrations of FAD.^[^ ^116^ ^]^

Regardless of the source of the fluorescent signal, fluorescence microscopy alone is unsuitable for clinical use in vivo due to the extensive dissection required for the access of conventional objectives to cochlear cells, including opening the temporal bone and otic capsule, which induce hearing loss. An alternative approach was used by Kim and Ricci in a study identifying the mechanism by which ototoxic aminoglycosides enter hair cells,^[^ ^117^ ^]^ where an imaging window was created by precisely thinning (to 80 microns) but not breaching the otic capsule using phosphoric acid gel and mechanical scraping. After injection of AM1‐43 dye through the round window to provide cellular contrast, this method permitted in vivo 2p cellular‐level imaging of the apical organ of Corti of mice.^[^ ^117^ ^]^ While the creation of the imaging window did not impair hearing, AM1‐43 is ototoxic and mice were deafened following injection. A similar imaging window could potentially be achieved via femtosecond laser ablation,^[^ ^118^ ^]^ but the window cannot be as thin as for brain imaging (10–30 microns) due to otic capsule fractures.^[^ ^117^, ^119^ ^]^ The potential for clinical translation is limited by the toxicity of injected AM1‐43 as well as the feasible placement and size of an imaging window in humans. The method could be adapted for the detection of FAD or other autofluorescence, although these will ultimately photobleach like all fluorescence signals. Further, the human otic capsule is substantially thicker than that of mice, requiring more thinning, and the working distance of air objectives may be insufficient to reach the human organ of Corti.

TPEF implementation into a fiber may be practical for intracochlear imaging via round window insertion. Specifically, one‐photon (1p) and 2p fluorescence imaging can be paired with compound gradient refractive index (GRIN) lenses within microendoscopes (350–1,000 µm in diameter) to provide sub‐cellular resolution and, with 2p imaging, optical sectioning of the tissue.^[^ ^120^ ^]^ The development and refinement of fluorescence microendoscopy has permitted imaging access to other deeply embedded brain structures unreachable by conventional optical microscopy.^[^ ^121^ ^]^ GRIN microendoscopes are rigid and have a short field of view (≈100 µm), necessitating close proximity to the imaged tissue and strictly maintained probe stability.^[^ ^120^ ^]^ Potential points of endoscopic access within the cochlea include a cochleostomy or the natural openings of the round window (≈1.8 width x 2.1 mm length) or oval window (≈1.26 × 2.40 mm).^[^ ^122^ ^]^ The diameters of these apertures place restrictions on the size of the endoscope that can be used for minimally‐invasive intracochlear imaging with the goal of hearing preservation. Rigid 1p fluorescence microendoscopy has been used to observe dynamic blood flow in the guinea pig cochlea in vivo, but required a cochleostomy.^[^ ^123^ ^]^ The resolution of 1.3 µm permitted the detection of single red blood cells within microvasculature, but hearing preservation was not assessed and is unlikely given the surgical approach. Flexible multi‐photon microendoscopy with high transverse (0.8 µm) and axial (12 µm) resolutions has been developed and used ex vivo on fixed human kidney or in vivo in mouse kidney,^[^ ^124^ ^]^ but has not been applied in the cochlea.

#### 2.2.5 Optical Coherence Tomography (OCT)

OCT is a cross‐sectional, natural contrast imaging technique that measures the intensity of backscattered or back‐reflected light from microstructural features at varying depths within biological tissues.^[^ ^125^ ^]^ OCT acquires high, 10–30 µm resolution, cross‐sectional images,^[^ ^125^ ^]^ and has been used to assess cochlear morphology and mechanics in animal models in vivo and ex vivo.^[^ ^8^, ^126^ ^]^ OCT's resolution is sufficient for detecting architectural features like the boundaries between the cochlea's fluid‐filled scalae,^[^ ^126^ ^]^ tectorial membrane,^[^ ^8^, ^126^, ^127^ ^]^ tunnel of Corti,^[^ ^8^ ^]^ and sensory epithelium,^[^ ^8^, ^126^ ^]^ but not individual cells or sub‐cellular features. OCT's capability to resolve the basilar membrane has enabled the measurement of high‐frequency mechanical vibrations in response to sound.^[^ ^126^, ^127^, ^128^ ^]^

OCT's penetration depth through bone of a few hundred microns^[^ ^129^ ^]^ permits imaging through the otic capsule of mouse (≈0.1 mm),^[^ ^130^ ^]^ but not of humans (1.8–3.1 mm).^[^ ^126^, ^131^ ^]^ This limitation can be overcome by pairing OCT with minimally‐invasive microendoscopy, which has been used to visualize the position of a CI electrode array in relation to the cochlear walls and structures in human temporal bones from organ donors.^[^ ^132^ ^]^ Additionally, a hand‐held OCT endoscope has been used to image the higher‐frequency areas of the cochlea through the round window via a myringotomy (incision in the tympanic membrane) in the middle ear of pigs.^[^ ^133^ ^]^ The ≈500 µm field of view permitted resolution of some soft tissue structures and measurement of sound vibrations, although there was low contrast for navigating the middle ear and the interior of the cochlea could not be imaged. Additionally, OCT faces challenges in resolving individual cells or sub‐cellular features due to limited resolution and speckle noise arising from coherent light detection.^[^ ^134^ ^]^

The development of micro‐OCT (µOCT), an advanced form of OCT with an order of magnitude better resolution of up to 1 µm,^[^ ^135^ ^]^ has helped to overcome these barriers. µOCT achieves superior axial resolution utilizing a very broad bandwidth source and common path interferometry, and lateral resolution using higher numerical apertures (NA) and extended depth of focus (EDOF) optics to illuminate the sample.^[^ ^135^, ^136^ ^]^ In extracted rodent cochleae, µOCT can visualize individual OHCs and Hensen's cells,^[^ ^136^ ^]^ including whether they are absent in noise‐damaged tissue.^[^ ^113^ ^]^ Further, 3D µOCT has enabled visualization of discrete outer pillar cells, the tectorial, basilar, and Reissner's membranes, and SGN fibers crossing the tunnel of Corti and space of Nuel to their OHC synapse regions.^[^ ^136^ ^]^ Previously, we paired µOCT imaging with a 600‐µm‐diameter, flexible intracochlear endomicroscope to provide high‐lateral‐resolution EDOF views in human cochleae, which had the required resolution to visualize individual cells and SGNs in the organ of Corti.^[^ ^137^ ^]^ This was implemented using self‐imaging wavefront division pioneered in other forms of catheter‐based µOCT.^[^ ^113^, ^138^ ^]^ The optical configuration focuses the beam to a small ≈2–4 µm diameter spot over ≈1 mm EDOF using a specially‐designed cylindrical waveguide proximal to the probe's focusing lens. The µOCT intracochlear endomicroscope's flexibility is equivalent to that of commercially available CIs and can be inserted into human scala tympani ex vivo over the entire first 360‐degree (basal) turn.^[^ ^137^ ^]^

While intracochlear µOCT holds promise for imaging sensorineural cells and nerves in patients due to its exceptional resolution, its contrast is based on tissue reflectance and scattering properties, relegating it to informing only on cochlear microstructure. However, further innovation of this technology has led to dynamic OCT (DOCT) and µOCT (DµOCT), which are capable of detecting both cytoarchitecture as well as ATP‐dependent intracellular motion, a proxy for cell viability and metabolic activity, without the use of fluorescent labels.^[^ ^139^ ^]^ During DOCT and DµOCT imaging, power spectrum data are separated to create pseudo‐colored composite images corresponding to the degree of intracellular movement (i.e., red, green, blue). Benchtop DOCT and DµOCT have been used to examine sub‐cellular structures and variations in cell metabolism in human esophageal and cervical biopsy samples,^[^ ^139^ ^]^ as well as in unfixed, unstained mammalian inner ears in two recent studies detailed below.^[^ ^140^ ^]^ Both studies used freshly excised mouse inner ears with the cochlea in situ and accessed the organ of Corti via an opening in the otic capsule.

**Figure 13:**
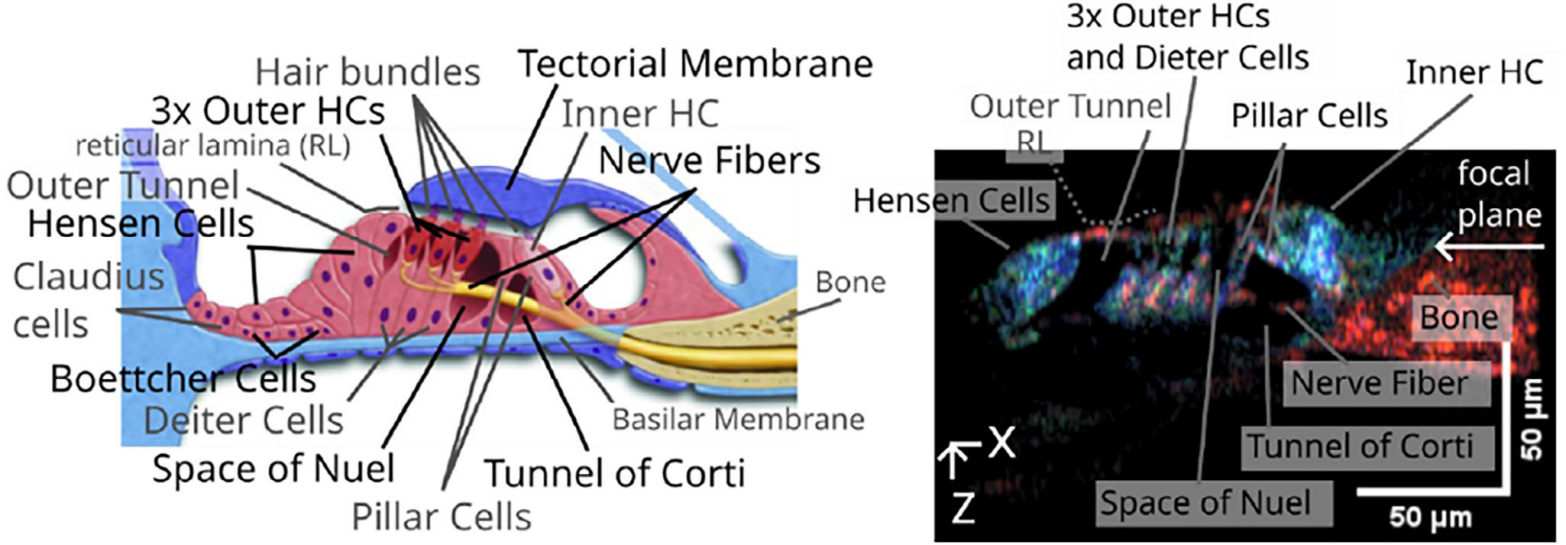
Dynamic optical coherence tomography of the basal region of the adult mouse organ of Corti (Atoh1TdT x TauGFP mice). A) Schematic of the organ of Corti with cells and anatomical features labeled. B) DOCT cross‐scan of a mouse organ of Corti ex vivo, with components color‐coded as red (0–2 Hz), green (2–5 Hz), and blue (5–62.5 Hz). The likely cells in the organ of Corti are identified, although cellular boundaries remain indistinct. Reproduced from Serafino et al. with permission.^[^ ^140^ ^]^ Copyright 2024, SPIE.

Serafino et al. imaged the organ of Corti of Atoh1TdT x TauGFP mice ex vivo using benchtop DOCT.^[^ ^140^ ^]^ The basal turn of the otic capsule was opened for imaging, and DOCT was successful in identifying bone, fluid‐filled spaces, membranes, and some cellular microanatomy. The pseudo‐coloring scheme used red to correspond to tissue motion between 0–2 Hertz (Hz; static), green to 2–5.0 Hz (high activity), and blue to 5.0–62.5 Hz (Brownian motion predominant). Notably, the resolution of DOCT was sufficient to distinguish the bone of the osseous spiral lamina (red in the right panel of **Figure** **13**), the sensory epithelium (blue‐green), and the tunnel of Corti and outer tunnel (black). Although degradation of lateral resolution rendered this method insufficient to distinguish the borders of individual hair cells and supporting cells with confidence, DOCT may nevertheless be sufficient depending on the clinical application.

**Figure 14:**
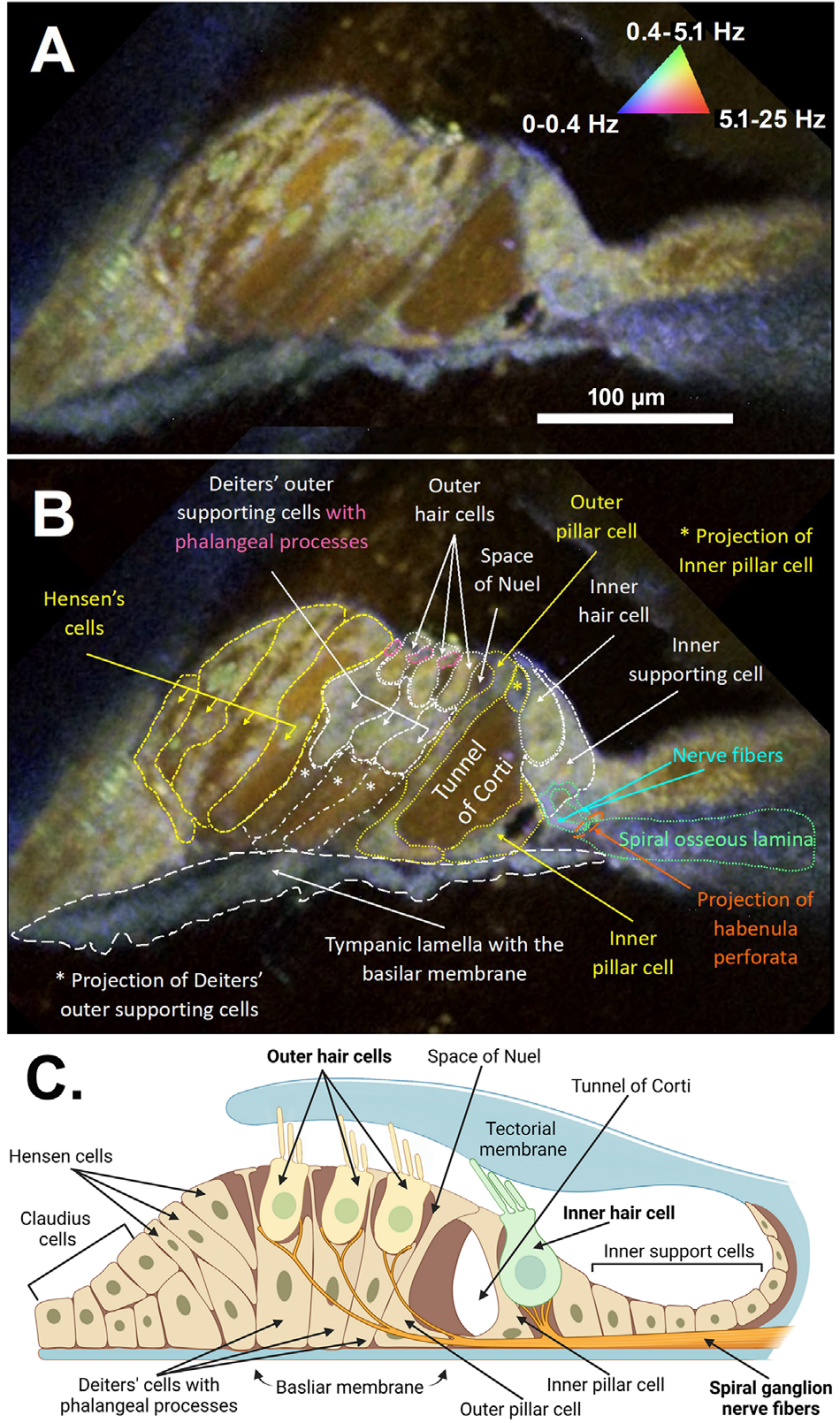
Dynamic micro‐optical coherence tomography (DµOCT) of the apical region of the adult mouse organ of Corti ex vivo without (A) and with (B) anatomical features annotated. Static anatomical features (e.g., basilar membrane circled in white dashes) appear blue, and Brownian motion‐predominant areas, such as the tunnel of Corti, appear brown‐red (triangle in A). Metabolically‐active cell features appear green‐yellow (nuclei, mitochondria, stereocilia). C) Schematic of the organ of Corti, including the inner and outer hair cells and non‐sensory cells. Reproduced from Schultz‐Hildebrandt et al.^[^ ^140^ ^]^ under the terms of the Creative Commons Attribution License (CC BY; https://creativecommons.org/licenses/by/4.0/). Copyright 2024, Frontiers Media S.A.

In Schulz‐Hildebrandt et al., a DµOCT benchtop system with a resolution of ≤2 µm was capable of resolving the microarchitecture of individual sensory and non‐sensory cells with higher detail, permitting discernment of cell boundaries, fluid‐filled lumens, and sub‐cellular structures such as nuclei (**Figure** **14**).^[^ ^140b^ ^]^ In this study, blue corresponded to tissue motion between 0–0.4 Hz (static), green to 0.4–4.9 Hz (metabolic activity), and red to 4.9–25 Hz (Brownian motion predominant).^[^ ^139^, ^141^ ^]^ Cell nuclei and hair cell stereocilia and cytoplasm appeared green‐yellow in the DµOCT image, indicating metabolic activity. Conversely, the static basilar membrane, reticular lamina, actin cytoarchitecture, and bone of the spiral osseus lamina appeared blue, while the fluid‐filled tunnel of Corti, space of Nuel, and supporting (Hensen, Deiters’, and Claudius) cells’ cytoplasm appeared red/brown, indicating Brownian motion. Notably, DµOCT could detect rapid shifts in cochlear cell metabolism and viability following the application of a cytotoxic insult (i.e., spectrum shifts from yellow/green to blue). As few as six frames, separated by 63 ms, were needed to reconstruct cochlear DµOCT images with sufficient detail to discern individual cells and assess their metabolic state.

Dynamic imaging with OCT holds promise for future clinical applications in otology and the unparalleled resolution may justify the use of surgically‐aided microendoscopy, contingent on the development of hearing preservation approaches. Although the finer cellular‐level detail achieved with DµOCT may be needed for single‐cell diagnostics, ultra‐high resolution or dynamic imaging may not necessarily be required. For example, the resolution of DOCT permits the ability to distinguish between the organ of Corti and bone, which may be sufficient to facilitate the placement of a CI electrode during surgical implantation. Thus, future research is needed to determine the most suitable clinical applications for these imaging methods.

## 3 Implications for Clinical Practice and Future Research

The sensorineural cells of the human cochlea are deeply embedded in the otic capsule and hard temporal bone, precluding non‐destructive biopsy to assess their status, and are 10–50 µm in size, placing them outside the resolution of conventional imaging modalities such as MRI, CT, and PET. Clinical light microscopes and rigid endoscopes routinely used to visualize the middle ear during otologic surgery similarly cannot provide insight into the defects of inner ear sensorineural cells due to lack of access, resolution, contrast, and optical sectioning.^[^ ^87^ ^]^ Further, the development of the optimal surgical approaches and delivery methods for therapy hinges on understanding the microanatomy and functionality of the human inner ear specifically. As a result of these limitations, there is currently no way to assess the unique cellular pathology that underlies each patient's SNHL in the otology clinic, precluding precision diagnosis and therapy selection.

As described in this review, several emerging imaging modalities have achieved cellular or near‐cellular resolution but are accompanied by other limitations that require further innovation to become viable clinical tools. SR‐PCI and 11.7T UHF‐MRI are non‐invasive and have improved resolution over conventional CT and MRI, respectively, but still fall below the level of cellular delineation, and the power required (i.e., as radiation or strong magnetic field) precludes in vivo use in humans. While fluorescence microscopy or microendoscopy provide cellular‐level detail, the requirement for very close access to cochlear cells, the invasive surgical approach to accommodate rigid endoscope optics, and the need for an applied or endogenous fluorescence signal have limited the in vivo applications for otology. Therefore, µOCT and DµOCT emerge as the most suitable new imaging modalities for translation due to their exceptional ability to resolve individual cells of the organ of Corti without sole reliance on fluorescence, provision of functional as well as anatomical information, and adaptability for flexible endoscopy that could better navigate complex cochlear anatomy and minimize trauma. µOCT methods could also be used to create 3D cochlear models (i.e., by using 3D printing) to enable patient‐level analysis of their unique anatomy, geometry, and sensory cell density along the length of the cochlea, which could be useful for CI fitting or probe design.

In this context, it is important to emphasize the crucial role of both basic and clinical science in advancing and validating novel cochlear imaging methodologies. Any laser‐based imaging modality requires rigorous safety studies to assess the risk of photonic or heat‐related tissue damage and to determine the ideal surgical approach to permit minimally invasive access to the cochlea while preserving hearing. The logical and least traumatic access point for future applications of DµOCT microendoscopy would be the round window in order to image the cochlea's basal turn. Since Von Ilberg et al.’s demonstration that a CI electrode could be introduced via the round window into the scala tympani while preserving residual hearing at lower frequencies,^[^ ^142^ ^]^ hearing‐preservation cochlear implantation has become routine. This is important as CIs are often indicated for patients with mid‐ and high‐frequency hearing loss who have serviceable acoustic hearing at lower frequencies. CI electrode placement is conventionally performed “blindly” or with low‐resolution endoscopic imaging of gross anatomy. However, high‐resolution intracochlear imaging could aid in the preservation of SGNs and optimal electrode placement important for the success of CIs.^[^ ^143^ ^]^ Currently, a “hearing preservation” approach to CI implantation uses atraumatic surgical techniques ensuring that the electrode array remains in the scala tympani and does not injure other structures such as the basilar membrane or osseous spiral lamina. The use of systemic corticosteroids, topical dexamethasone, or topical hyaluronic acid has also been shown to minimize cochlear trauma during implantation,^[^ ^144^ ^]^ and could potentially also be used prior to microendoscopy procedures. Further, basic science investigations are essential for expanding our understanding of the anatomy, fluid dynamics, genetics, pathology, and regenerative potential of the cochlea which, compared to many other organs, remain nascent. As long as the cochlea remains a “black box,” otologists and their patients cannot benefit from fit‐to‐purpose imaging methodology and instead must try to adapt existing approaches poorly suited to this unique organ.

It is difficult to overstate the impact of medical imaging on improving patient outcomes by enabling earlier disease detection, routine screening, precision diagnosis, and visualization of surgical approaches.^[^ ^145^ ^]^ The advent of routine X‐ray‐based mammography has reduced breast cancer mortality by 41% and the rate of advanced breast cancer by 25%.^[^ ^146^ ^]^ Similarly, endoscopy‐enabled colonoscopy and sigmoidoscopy have significantly reduced colorectal cancer mortality.^[^ ^147^ ^]^ MRI and CT have both been instrumental in reducing mortality and guiding treatment for head injury and ischemic stroke, including the identification of “silent strokes,” which can greatly impair cognitive functioning.^[^ ^148^ ^]^ Although SNHL, as a discrete disorder, is not fatal, it remains a leading sensory disability and profoundly impacts multiple facets of a patient's life, as well as society overall. Novel therapies tailored for specific forms of hearing loss are greatly needed to address these burdens, yet there are currently no pharmaceutical, gene, or cell therapies approved in the US for the treatment of the underlying causes of SNHL.^[^ ^149^ ^]^

The lack of high‐resolution imaging of the human inner ear has presented major challenges for the development and assessment of therapies for hearing loss. For example, severe or prolonged insults can render the organ of Corti a flat epithelium devoid of sensory cells,^[^ ^24^ ^]^ which has resisted *ATOH1* gene therapy,^[^ ^150^ ^]^ but could be amenable to stem cell therapy.^[^ ^151^ ^]^ “Dead” regions in the organ of Corti are believed to underlie specific frequency deficits,^[^ ^152^ ^]^ which could enable targeted therapy if identified. Some SNHL is due to stereocilia or synaptic defects, although the hair cells themselves may remain functional.^[^ ^153^ ^]^ Recently, gene therapy for otoferlin mutations is being tested in open‐label clinical trials, and early results illustrate the transformative potential of this approach.^[^ ^25^ ^]^ However, otoferlin gene therapy requires patients to have this rare mutation as well as functional remaining hair cells, limiting its applicability to a small population. Patients with otoferlin mutations can experience post‐natal degeneration of their hair cells,^[^ ^154^ ^]^ and having a tool to assess cellular structure and functionality during screening for therapeutic candidacy would be invaluable, complementing otoacoustic emissions, which reflect the function of OHCs only. Given this breakthrough in therapy, which will hopefully spur additional novel SNHL treatments, the present moment is ideal for accelerating the development of human in vivo intracochlear imaging.

## 4 Conclusion 

The potential therapeutic applications, as well as opportunities to enable scientific insight, underscore the need to visualize both the microstructure and viability of the sensorineural cells of the human organ of Corti. Although currently available clinical imaging modalities have insufficient resolution for cellular‐level imaging of the cochlea, emerging high‐resolution methodologies such as OCT and DµOCT hold enormous promise for overcoming these obstacles. Inspired by the historical progress in surgical imaging paved by otologists Nylén and Holmgren, we anticipate that high‐resolution microstructural and functional imaging of the inner ear will similarly catalyze progress across medicine and surgery.

## Conflict of Interest

G.T. and K.S. are inventors on patents pertaining to µOCT and DµOCT technology and have received sponsored research funding from WayVector. S.B. and N.P. have no affiliations, memberships, funding, or financial holdings that might be perceived as affecting the objectivity of this review.

## Ethics Approval Statement

The images in Figures 3, 4, 5, 6, 7, 8, and 11A, and Supplemental Videos 2‐4, were derived from patients treated at Stanford University School of Medicine. The retrospective use of these de‐identified images was approved by the Human Subjects Institutional Review Board of Stanford University (IRB‐71759).

## Supporting information



Supplemental Video 1: 3D rotational model of the human inner ear. This model was created from archival histological sections from a 14‐year‐old human male. The specimen was formalin fixed, decalcified, embedded in celloidin, serially sectioned in the axial plane at 20 microns, stained with hematoxylin and eosin, and mounted on slides. Low‐power views of every fifth section through the temporal bone were digitized and imported into Amira v3.1 (Mercury Computer Systems/TGS, San Diego, CA). The sections were aligned and segmented into anatomical structures of interest. Light transparent pink, cochlea and vestibular system. Yellow, vestibular branch of cranial nerve VIII. Light green, auditory branch of cranial nerve VIII. Magenta, endolymphatic lumen (housing the organ of Corti) and duct. White, vestibular end organs (saccule, utricle, and ampulla of the semicircular canals). Orange, round window membrane. The video was created with the EPL 3‐D Surface Viewer of Human Temporal Bone v2.0a1. The software was developed under the supervision of Drs. M. Charles Liberman and Saumil Merchant by Haobing Wang of Eaton‐Peabody Laboratory of Auditory Physiology, Massachusetts Eye and Ear, supported by a core grant from the NIDCD (P30 DC05209). Available at: https://www.masseyeandear.org/research/otolaryngology/eaton‐peabody‐laboratories/temporal‐bone‐model.

Supplemental Video 2: 3D Computed tomography (CT) of normal human temporal bone.

Supplemental Video 3: Magnetic resonance imaging (MRI) of inner ear structures (axial). Heavily T2‐weighted cine images demonstrating the normal fluid‐filled inner ear structures.

Supplemental Video 4: Magnetic resonance imaging (MRI) of inner ear structures (coronal). Heavily T2‐weighted cine images demonstrating the normal fluid‐filled inner ear structures.
